# The Cycloaddition of the Benzimidazolium Ylides with Alkynes: New Mechanistic Insights

**DOI:** 10.1371/journal.pone.0156129

**Published:** 2016-05-25

**Authors:** Costel Moldoveanu, Gheorghita Zbancioc, Dorina Mantu, Dan Maftei, Ionel Mangalagiu

**Affiliations:** Department of Chemistry, Alexandru Ioan Cuza University of Iasi, Iasi, Romania; University of Sydney, AUSTRALIA

## Abstract

New insights concerning the reaction mechanism in the cycloaddition reaction of benzimidazolium ylides to activated alkynes are presented. The proposed pathway leading both to 2-(1H-pyrrol-1-yl)anilines and to pyrrolo[1,2-a]quinoxalin-4(5H)-ones involves an opening of the imidazole ring from the cycloaddition product, followed by a nucleophilic attack of the aminic nitrogen to a proximal carbonyl group and the elimination of a leaving group. The mechanistic considerations are fully supported by experimental data, including the XRD resolved structure of the key reaction intermediate.

## Introduction

**Fig 1:**
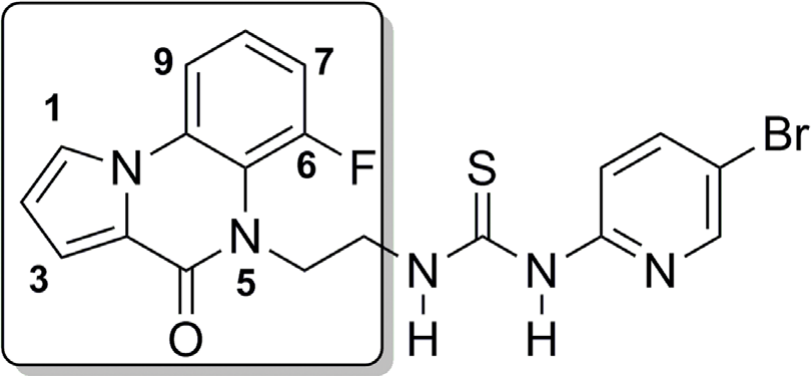
Structural formula of 6-FQXTP.

Pyrrolo[1,2-a]quinoxalinone derivatives are an important class of heterocyclic compounds due to their biological activities. Some carboxylic acid derivatives of pyrrolo[1,2-a]quinoxalin-4(5H)-one show significant (about 100 times larger than disodium cromoglycate) antiallergic activity in the passive cutaneous anaphylactic (PCA) test following either, and in some cases both, intravenous or oral dosing [1]. Moreover, the quinoxaline system has been identified as a critical structural requirement for optimal interaction with the human immunodeficiency virus type 1 (HIV-1) non-nucleoside reverse transcriptase inhibitors (NNRTI) binding site [2]. 6-Fluoro-quinoxalinylethylpyridylthiourea (6-FQXTP, Fig 1) represent the prototype of this class of NNRTI [2].

Few methods are available for the synthesis of the pyrrolo[1,2-a]quinoxalinone. One method requires expensive palladium catalyst for an intramolecular carbon-nitrogen bond formation [3]. Another method requires copper-catalyzed intramolecular *N*-arylation of the Ugi four component reaction product of aldehydes, 2-iodoaniline, 2-indole carboxylic acids, and isocyanides in one-pot procedure [4]. Cycloelimination of ammonia by anhydrous tin (II) chloride–hydrochloric acid reduction of the pyridazinoquinoxalinones [5], gives the same pyrrolo[1,2-a]quinoxalinone derivatives. The pyrrolo[1,2-a]quinoxalinone scaffold was also obtained by 1,3-dipolar cycloaddition of non-stabilized pyridinium methylides to dipolarophiles [6]. The non-stabilized pyridinium methylides were generated from *N*-(silylmethyl)pyridine analogs via 1,4-silatropy [6]. The *N*-substituted-1-(2-nitrophenyl)-1*H*-pyrrole-2-carboxamides undergo denitrocyclisation with NaH in DMF to the corresponding 5-alkyl(or aryl) pyrrolo[1,2-a]quinoxalin-4(5*H*)-ones [7]. A widely used, but also disputed method for the synthesis of the pyrrolo[1,2-a]quinoxalinone derivatives is the cycloaddition reaction of the benzimidazolium ylides to dipolarophyles [8–15]. Among the first uses, the works of Ogura [12] report the formation, along the pyrrolo[1,2-a]benzimidazoles, of either 2-propenylidene-benzimidazolines (in the case of benzimidazolium ylides derived from acetophenones), or 1-oxo-1,5(2H)-pyrido[1,2-a]benzimidazoles (when using benzimidazolium ylides derived from halogeno-esters). The authors explain the formation of both byproducts by the aromatization of the imidazole ring concomitant with the opening of pyrrole ring, while in the case of the latter the subsequent elimination of a methoxy group is required. A few decades later, Zhang and Huang [9,10] isolated a pyrrolo[1,2-a]quinoxaline instead of the expected pyrrolo[1,2-a]benzimidazoles, the mechanism proposed to explain the finding involving a concerted ring expansion, and hence no intermediates. Recent highlights [11,16,17] show that, depending on the substituents and/or the conditions employed, the reaction may be tuned toward the formation of either pyrrolo[1,2-a]benzimidazoles, pyrrolo[1,2-a]quinoxalin-4-ones [16] or pyrrolo[1,2-a]quinoxalines [11]. The mechanism proposed involves aromatization of the pyrrole ring concomitant with the opening of imidazole ring leading to 2-pyrrolo-aniline intermediates. Intermediates bearing good leaving groups in the 2nd position of the pyrrole ring allow further elimination to give pyrrolo[1,2-a]quinoxalin-4-ones, whereas a non-leaving group leads to pyrrolo[1,2-a]quinoxalines. However, no intermediate was isolated in any of the previous works.

In view of the above consideration and our background in the cycloimmonium ylides area [18–29], we decided to investigate the cycloaddition reaction of the benzimidazolium ylides, with dimethylacetylene dicarboxylate (DMAD) as dipolarophile in order to elucidate the reaction mechanism.

## Materials and Methods

### Apparatus and analysis

All reagents and solvents were purchased from commercial sources and used without further purification. Melting points were recorded on a MEL-TEMP II apparatus in open capillary tubes and are uncorrected. Analytical thin-layer chromatography was performed with commercial silica gel plates 60 F_254_ (Merck) and visualized under UV light. The NMR spectra were recorded on a Bruker Avance III 500 MHz spectrometer operating at 500 MHz for ^1^H and 125 MHz for ^13^C. Infrared (IR) data were recorded as films on potassium bromide (KBr) pellets on a FT-IR Shimadzu Prestige 8400s spectrophotometer. The X-Ray diffraction experiment was performed using a SuperNova Dual diffractometer equipped with a Cu (Kα radiation, λ = 0.684 Å) fine-focus sealed X-ray tube and a graphite monochromator. Detector resolution: 16.1593 pixels mm^-1^. Absorption correction: multi-scan (CrysAlis PRO; Agilent, 2011), Tmin = 0.914, Tmax = 1.000. The reflections were recorded at room temperature on a small single crystal.

### Typical procedure for the cycloaddition reaction of the benzimidazolium ylides with DMAD

A mixture of benzimidazolium salts **1a-i** (3 mMol) and DMAD (0.852 g, 6 mMol) was suspended in 15 mL chloroform. Then, triethylamine (0.606 g, 6 mMol) dissolved in 10 mL chloroform was added drop wise under stirring in one hour. The stirring and refluxing were continued for 12 hours. After the reaction was finished (TLC), the obtained solution was cooled down at room temperature and then the reaction mixtures was washed with water (3 x 30 mL), dried over magnesium sulfate and evaporated under reduced pressure to give the crude product. The purification of the crude product was done by column chromatography on silica gel (eluted with CH_2_Cl_2_ to 98/2 CH_2_Cl_2_/ CH_3_OH) giving either two products in the cases of the **1a** and **1b** salts or a single product in the other cases.

## Results and Discussion

**Fig 2:**
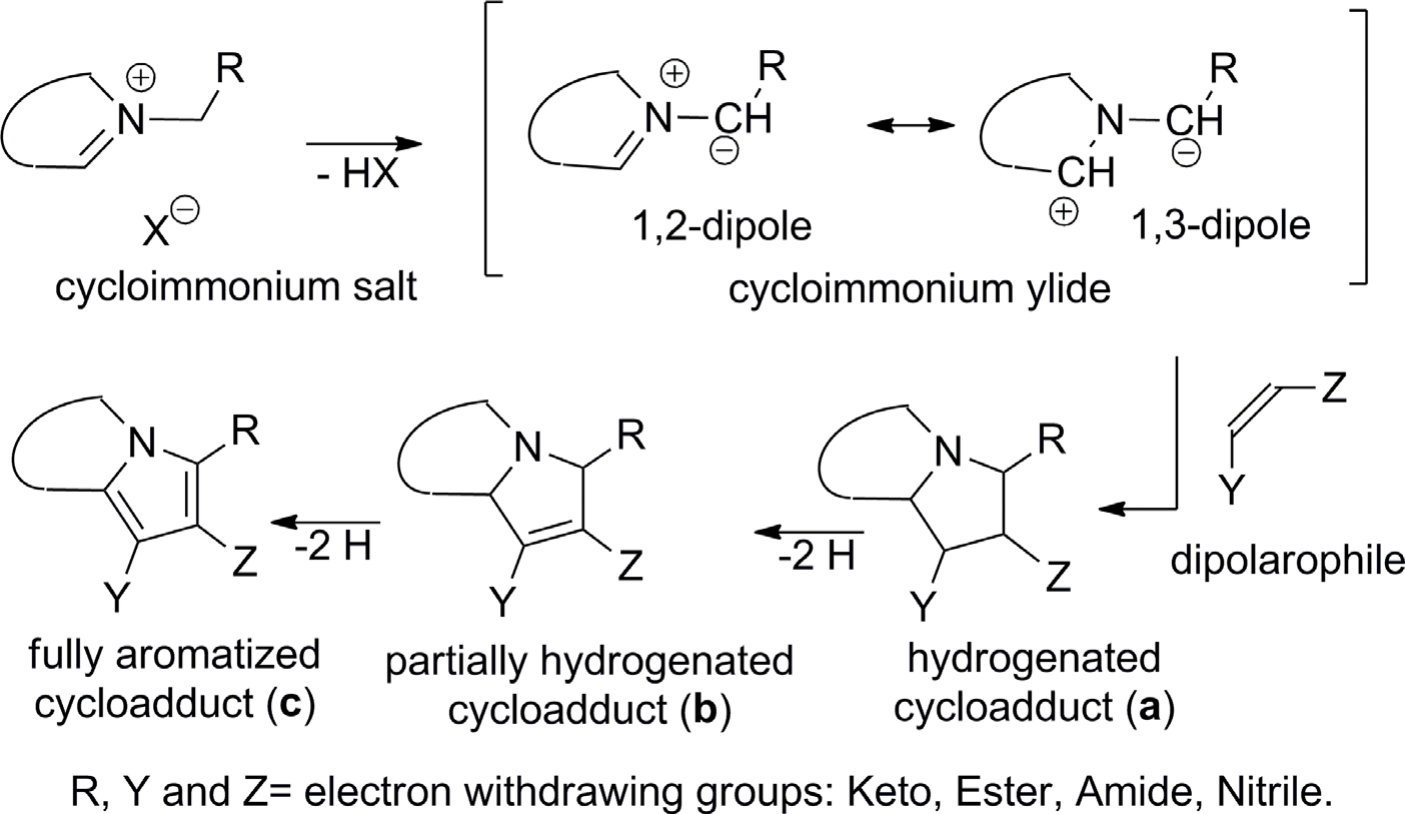
Reaction pathway for the cycloaddition of cycloimmonium salts to olefinic dipolarophiles.

The cycloaddition reaction of cycloimmonium ylides involves three stages: (i) generation of the ylide from the corresponding salt; (ii) a Huisgen 3+2 cycloaddition of ylide to dipolarophile, with the formation of a cycloadduct; (iii) total or partial dehydrogenation of the intermediary cycloadduct, with the final formation of a thermodynamically more stable aromatized adduct (Fig 2). The first two stages have been thoroughly investigated and described in literature [30,31], while the intermediate’s dehydrogenation in the third stage leads to a large variety of products including total or partial hydrogenated [21,23], fully aromatized cycloadducts [18–29] or even with an altered structure of the cycloadduct [8,9,12,14]. It is the latter case we focus in the following.

**Fig 3:**
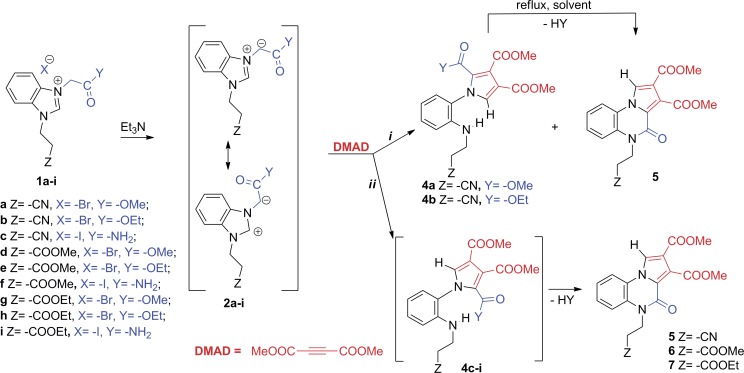
Reaction pathway for the cycloaddition reactions of benzimidazolium ylides to DMAD.

In order to rationalize the literature data, to elucidate the reaction mechanism and to obtain new pyrrolo[1,2-a]quinoxalinone derivatives, we decide to study the reactions of benzimidazolium ylides (generated *in situ*, using triethylamine, from the corresponding salts **1a-i** [27–29]) with the activated alkyne, DMAD, (Fig 3).

In contrast to literature data [12–14,16,17,19,21,26], in our case we did not isolate any hydrogenated (type **a**), partially hydrogenated (type **b**) or fully aromatized cycloadduct (type **c**), see Fig 2.

Instead, in the case of ylides **2a**,**b**, two types of stable products were obtained and isolated: 2-(1H-pyrrol-1-yl)anilines (**4a** and **4b)**, and pyrrolo[1,2-a]quinoxalin-4(5H)-one **5** (Fig 3, pathway ***i***). In the case of the other ylides **2c-i** only pyrrolo[1,2-a]quinoxalin-4(5H)-ones **5**, **6** and **7** were isolated, whereas the NMR spectra of the crude products indicate the presence of the 2-(1H-pyrrol-1-yl)anilines **4c-i** as unstable intermediates, which in time stabilize to quinoxaline derivatives **5**, **6** and **7** (Fig 3, pathway ***ii***).

**Fig 4:**
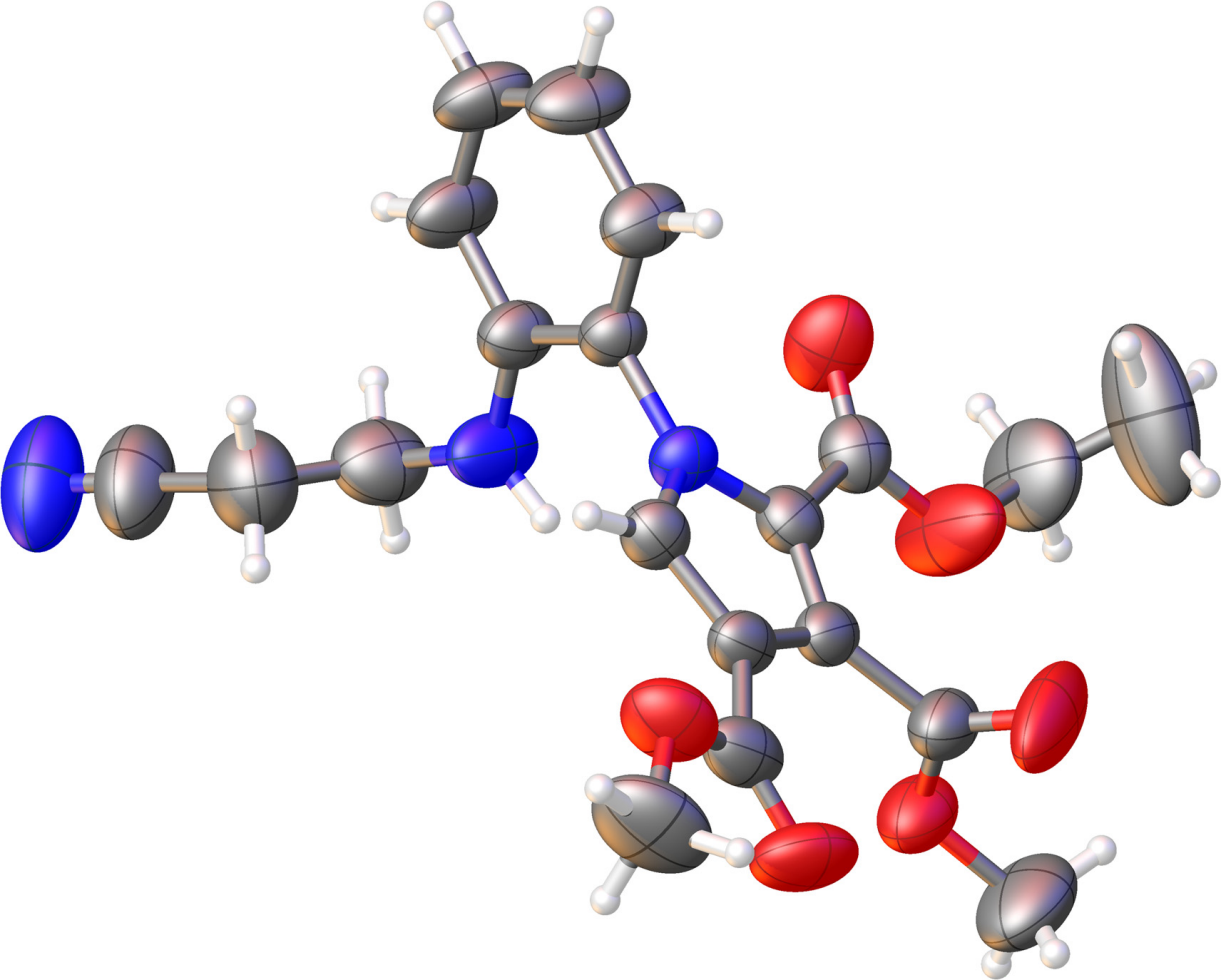
ORTEP representation at 50% probability for compound 4b.

**Fig 5:**
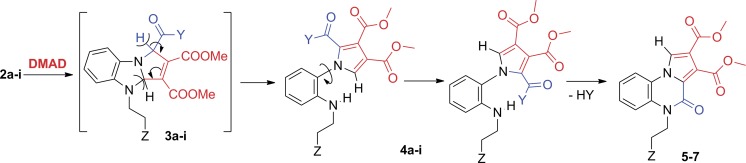
Proposed mechanism for the cycloaddition reactions of benzimidazolium ylides to DMAD.

XRD resolved structure of an isolated reaction intermediate (**4b**, Fig 4) suggests that after the initial formation of the cycloaddition products with a dihydropyrrolo[1,2-a]benzimidazole structure (**3a-i**), the reaction mechanism (Fig 5) involves a ring opening of the imidazole cycle [10,11,16,17] (and not of the pyrrole ring as proposed previously in the literature [12]) with the formation of a conformer of the 2-(1H-pyrrol-1-yl)anilines **4a-i**.

The formation of compounds **4a,b** may be assisted by the abstraction of the hydrogen atom from the acidic α-position of the ester/amide Y in the presence of excess triethylamine with concomitant fragmentation of the imidazole ring and aromatization of the pyrrole ring. Twisted conformations of the resulting 2-(1H-pyrrol-1-yl)anilines **4a-i** (76° in the case of **4b**) arise from a free rotation of the pyrrole ring around the N_pyrrole_-C_aryl_ single bond. According to the nature of Z and Y substituents, the amines **4a-i** are either stable (compounds **4a,b**) or unstable (compounds **4c-i**), a cyclization process to the six-membered ring of pyrrolo[1,2-a]quinoxalin-4(5H)-one structure (**5–7**) taking place in case of the latter, via elimination of an alkoxy group, Y (Fig 3, pathway ***ii***). Given the tendency of spontaneous cyclization observed in the case of amines **4c-i**, our next attempt was to convert the amines **4a,b** into a corresponding pyrrolo[1,2-a]quinoxalin-4(5H)-one structure, expected to be thermodynamically more stable. Indeed, the desired pyrrolo[1,2-a]quinoxalin-4(5H)-one **5** was easily obtained from both **4a** and **4b** cases by reflux in solution (Fig 3). Conversion of the two amines to the corresponding quinoxalinone also occurs at room temperature, in solution. Results of ^1^H NMR studies on **4b** at room temperature (see S6 Fig) reveal that intramolecular cyclization is a slow process, spectra recorded after 11 days from sample preparation containing signals from both **4b** and **5** in nearly equimolar ratio.

The isolation of the 2-(1H-pyrrol-1-yl)anilines **4a,b** is the missing link that confirms the mechanism proposed by Georgescu [11,16,17] and in the same time infirms the concerted mechanism proposed by Zhang and Huang [9,10].

A summary of the products yielded through the cycloaddition of the benzimidazolium ylides to DMAD are listed in Table 1.

**Table 1:** Yields and products obtained in the cycloaddition reactions of benzimidazolium ylides 2a-i.

Salt	Z	X	Y	2-(1H-pyrrol-1-yl)anilines	Yield (%)	pyrrolo[1,2-a]quinoxalin-4(5H)-ones	Yield (%)
**2a**	CN	Br	OMe	**4a**	19	**5**	19
**2b**	CN	Br	OEt	**4b**	19	**5**	12
**2c**	CN	I	NH_2_	**4c**	-^a^	**5**	10
**2d**	CO_2_Me	Br	OMe	**4d**	-^a^	**6**	39
**2e**	CO_2_Me	Br	OEt	**4e**	-^a^	**6**	23
**2f**	CO_2_Me	I	NH_2_	**4f**	-^a^	**6**	15
**2g**	CO_2_Et	Br	OMe	**4g**	-^a^	**7**	26
**2h**	CO_2_Et	Br	OEt	**4h**	-^a^	**7**	26
**2i**	CO_2_Et	I	NH_2_	**4i**	-^a^	**7**	10

Yields given in lines 1 and 2 are additive (it was a mixture of 4 + 5 with the individual yields of the two components given after their column chromatography separation from the product mixture ΔRf = 0.15 on 97/3 CH_2_Cl_2_/ CH_3_OH)

^a^Not isolated, observed in NMR of the crude reaction mixture. Reaction conditions: benzimidazolium salts: DMAD: triethylamine = 1:2:2; then refluxing for 12 hours in chloroform

One may note from Table 1 that the isolated yields are low to moderate, in good agreement with values reported in the literature for this type of reactions. The lower yields in the case of the benzimidazolium ylides **2c**, **2f** and **2i** should relate to a different behaviour of the NH_2_ as leaving group compared to the alkoxy counterpart, that impacts in case of the former on cyclization to quinoxaline ring.

**Fig 6:**
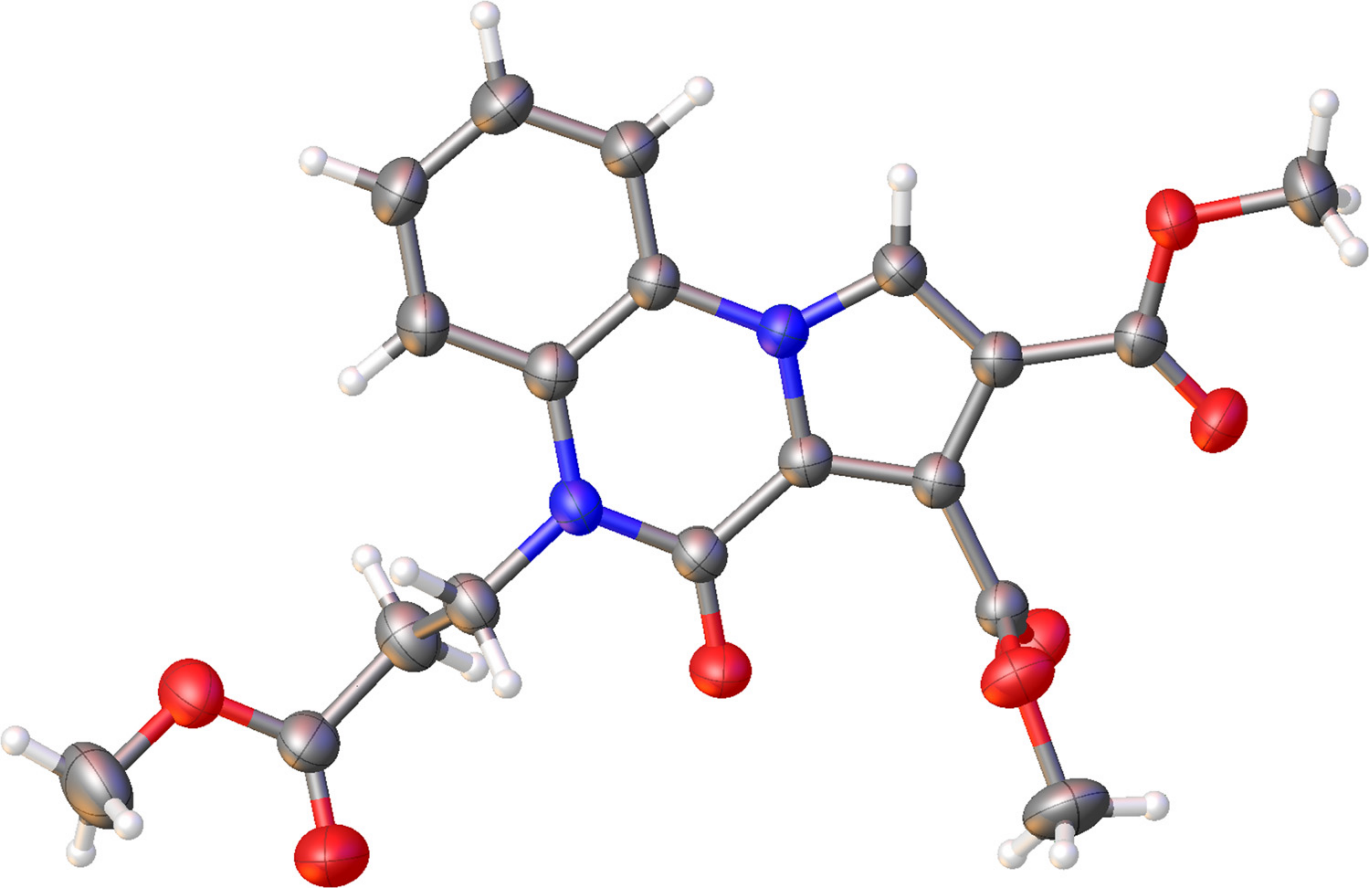
ORTEP representation at 50% probability for compound 6.

The structures of cycloaddition products **4a-i, 5, 6, 7** were determined unambiguously by spectral analysis (IR, ^1^H-NMR, ^13^C NMR, and two-dimensional experiments 2D-COSY, 2D-HETCOR (HMQC), long range 2D-HETCOR (HMBC) and finally, in the case of 2-(1H-pyrrol-1-yl)anilines **4b**, and pyrrolo[1,2-a]quinoxalin-4(5H)-one **6** also by single crystal X-ray diffraction (Figs 4 and 6 respectively).

## Conclusions

Our results reported herein complement previously reported literature data regarding the cycloaddition of benzimidazolium ylides to dipolarophiles, adding new insights into the reaction mechanism. A plausible explanation for obtaining both types of cycloaddition products is provided by the reaction mechanism and correlates to the literature data. The proposed pathway leading both to 2-(1H-pyrrol-1-yl)anilines and to pyrrolo[1,2-a]quinoxalin-4(5H)-ones, involving the opening of the imidazole ring, is supported by spectral analysis and X-ray diffraction experiments. We also shown that reaction selectivity toward pyrrolo[1,2-a]quinoxalin-4(5H)-ones may be tuned by experimental conditions.

## Supporting Information

S1 Cif Crystallographic Information Files (CIF) of the compound 4b. (CIF)

S2 Cif Crystallographic Information Files (CIF) of the compound 6. (CIF)

S1 Fig **(a)** ^1^H NMR spectrum of the compound **4a.(b)** ^13^C NMR spectrum of the compound **4a.**
(TIF)

S2 Fig **(a)** ^1^H NMR spectrum of the compound **4b.(b)** ^13^C NMR spectrum of the compound **4b.**
(TIF)

S3 Fig **(a)** ^1^H NMR spectrum of the compound **5.(b)** ^13^C NMR spectrum of the compound **5.(c)** Detail on ^13^C NMR spectrum of the compound **5.**
(TIF)

S4 Fig **(a)** ^1^H NMR spectrum of the compound **6.(b)** ^13^C NMR spectrum of the compound **6.**
(TIF)

S5 Fig **(a)** ^1^H NMR spectrum of the compound **7.(b)** ^13^C NMR spectrum of the compound **7.**
(TIF)

S6 Fig Time evolution of the ^1^H-NMR spectrum of 2-ethyl 3,4-dimethyl 1-(2-(2-cyanoethylamino)phenyl)-1H-pyrrole-2,3,4-tricarboxylate (4b). (TIFF)

S1 File Supporting Information document. Spectral characterization, NMR spectra (^1^H and ^13^C) of the obtained compounds, and ^1^H-NMR studies on **4b** at room temperature.
(DOCX)
