# Supplementary figures and images for: The Cycloaddition of the Benzimidazolium Ylides with Alkynes: New Mechanistic Insights
Source: PLoS One. 2016 May 25;11(5):e0156129. doi: 10.1371/journal.pone.0156129 (PMC4880325; doi:10.1371/journal.pone.0156129)

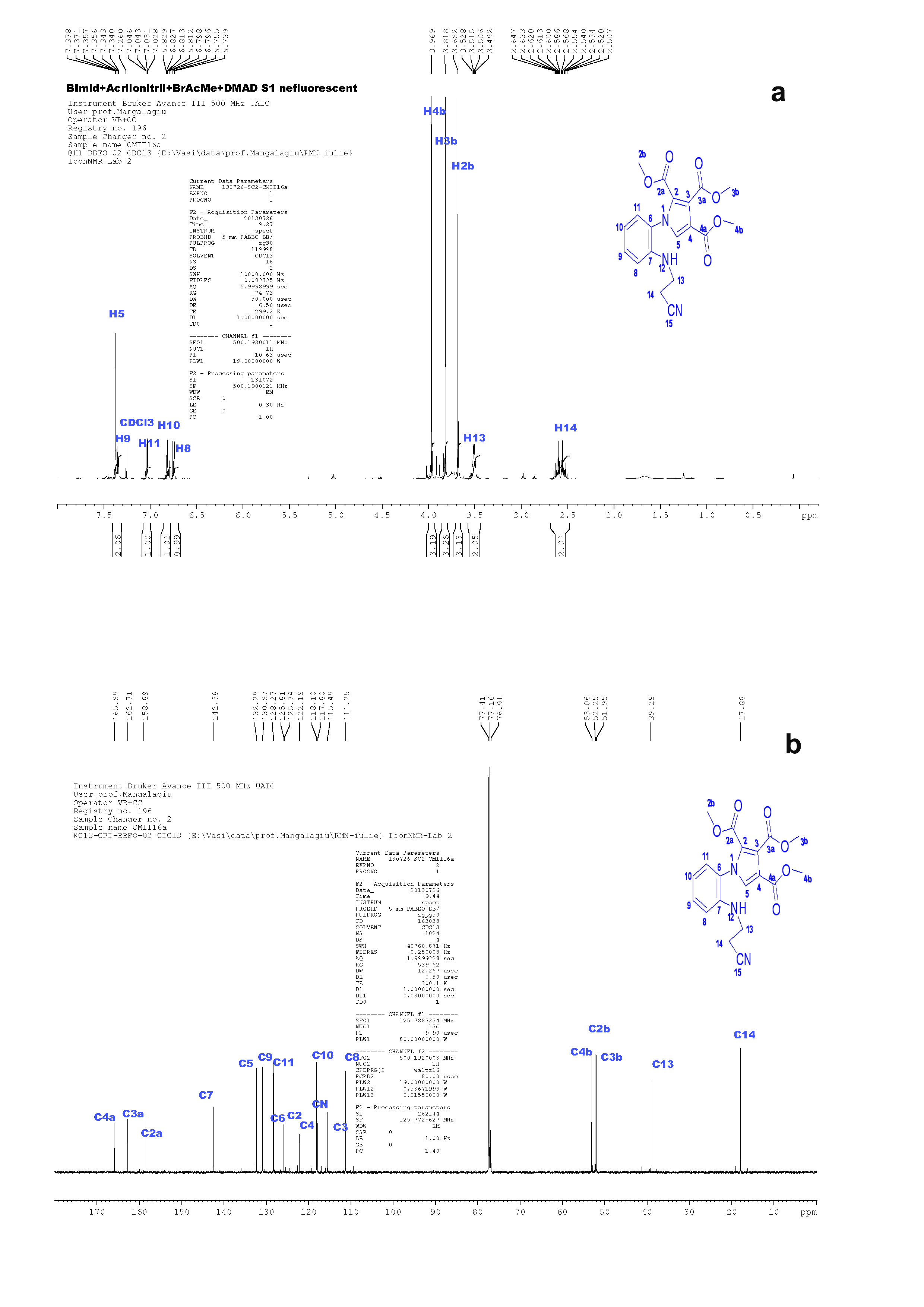

Supplement: S1 Fig — (a) 1H NMR spectrum of the compound 4a.(b) 13C NMR spectrum of the compound 4a. (TIF) [file pone.0156129.s003.tif]

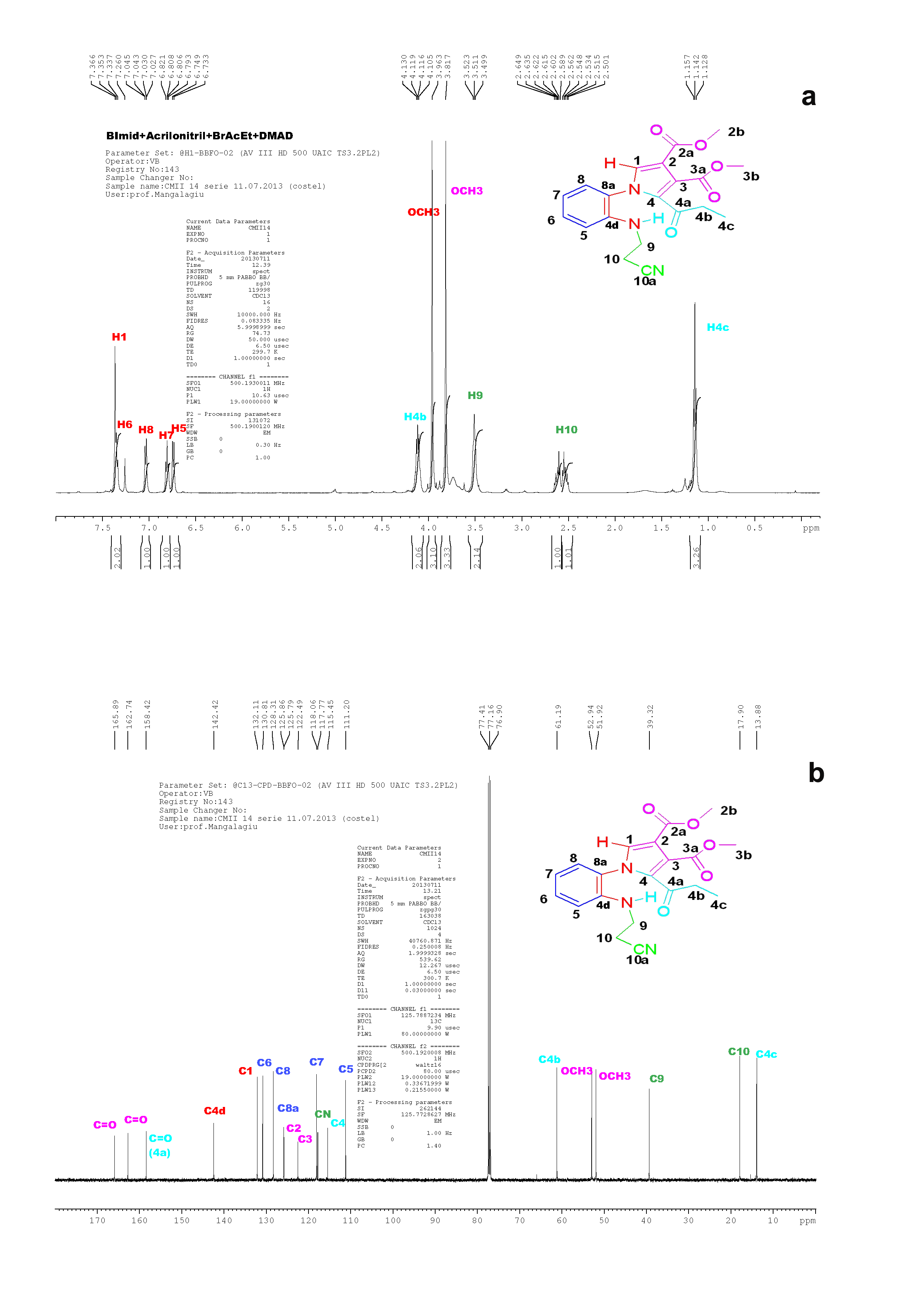

Supplement: S2 Fig — (a) 1H NMR spectrum of the compound 4b.(b) 13C NMR spectrum of the compound 4b. (TIF) [file pone.0156129.s004.tif]

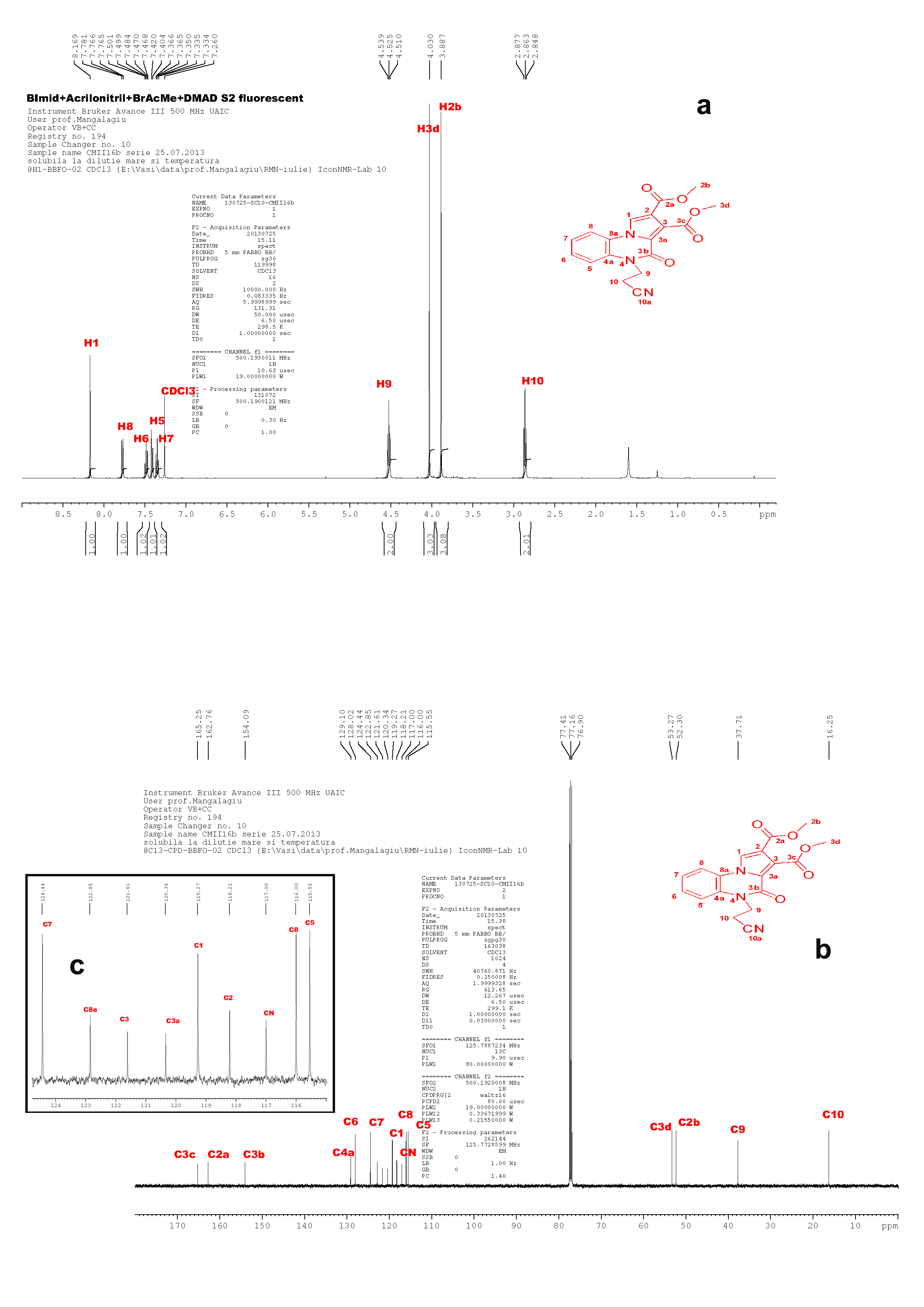

Supplement: S3 Fig — (a) 1H NMR spectrum of the compound 5.(b) 13C NMR spectrum of the compound 5.(c) Detail on 13C NMR spectrum of the compound 5. (TIF) [file pone.0156129.s005.tif]

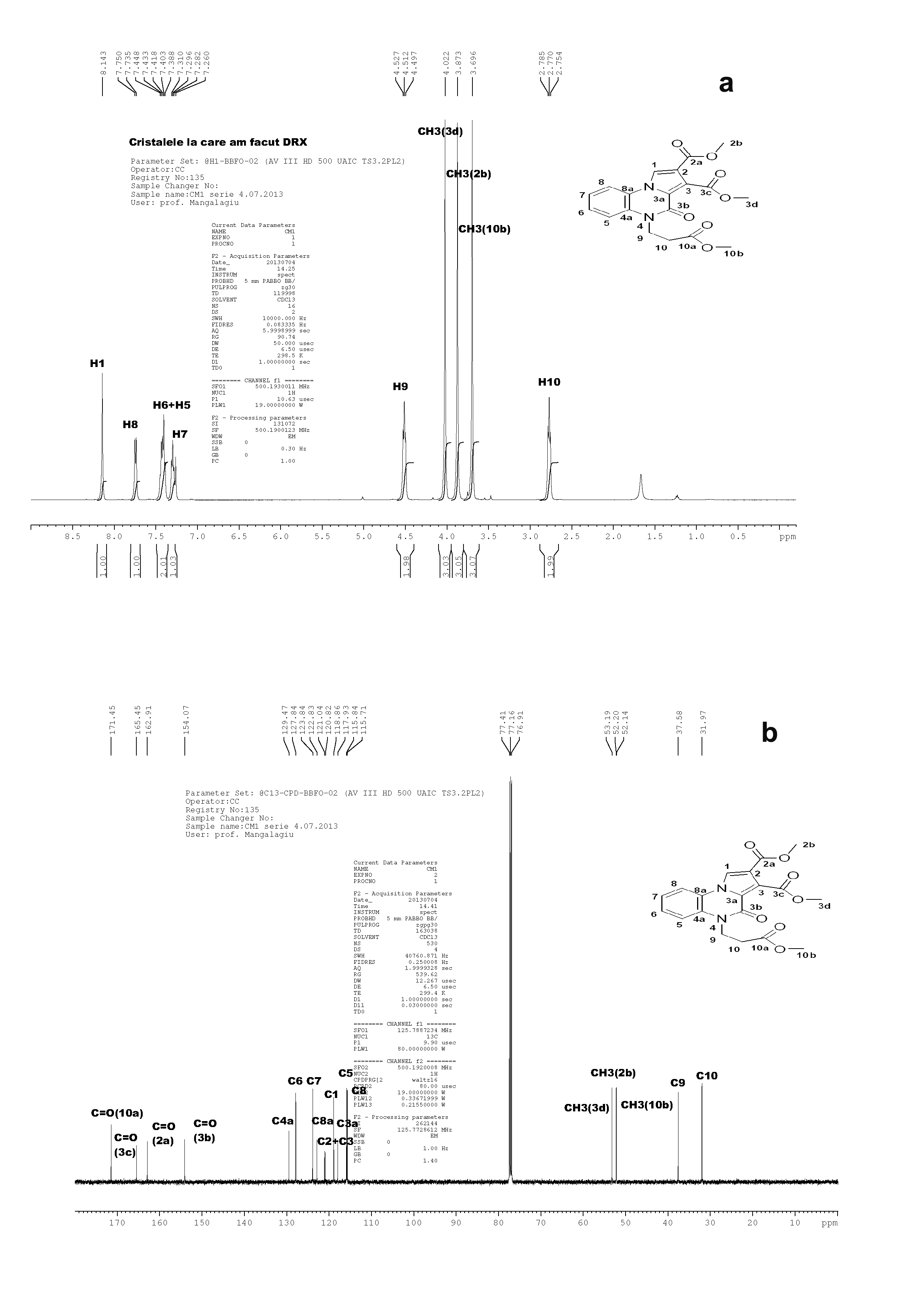

Supplement: S4 Fig — (a) 1H NMR spectrum of the compound 6.(b) 13C NMR spectrum of the compound 6. (TIF) [file pone.0156129.s006.tif]

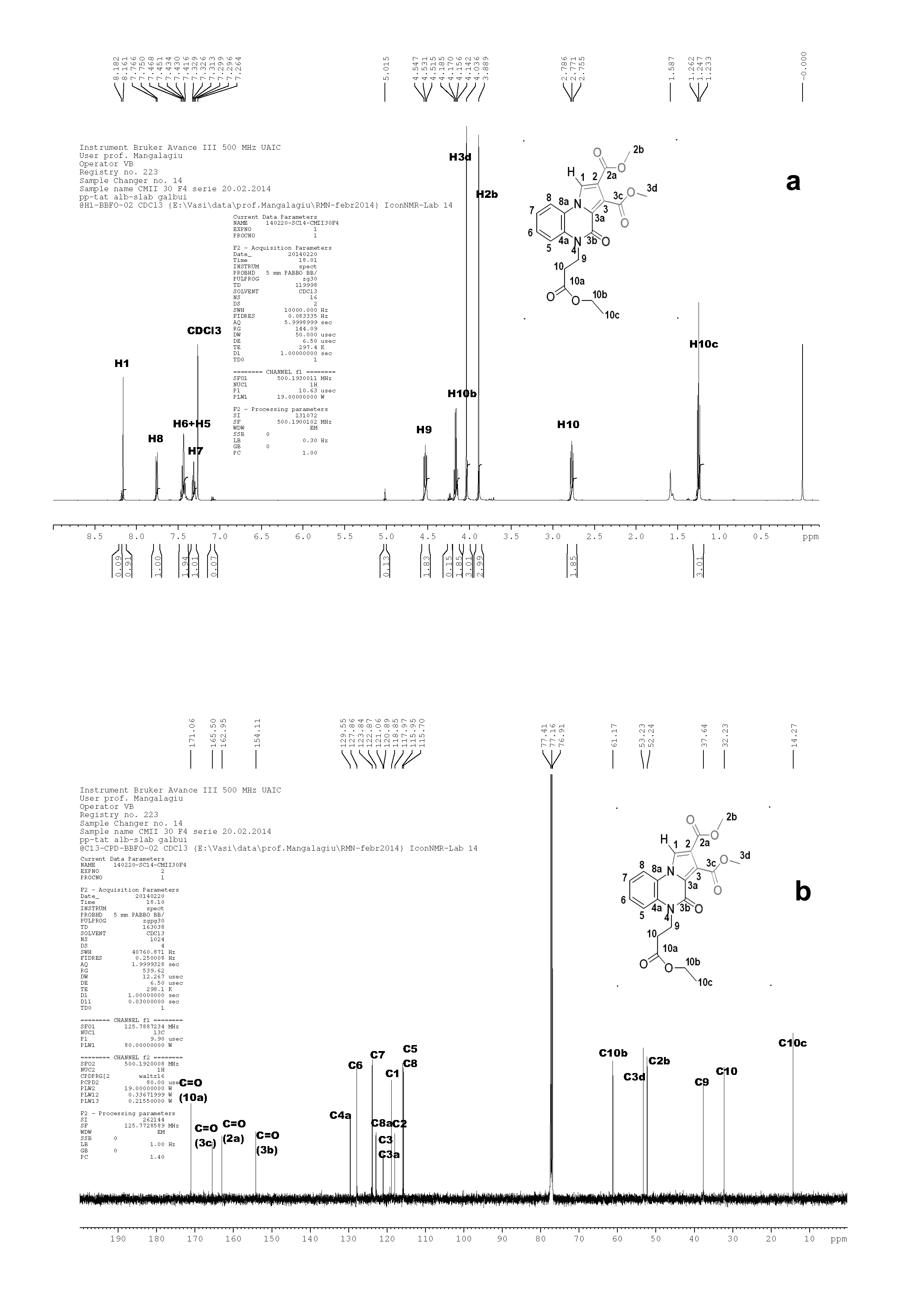

Supplement: S5 Fig — (a) 1H NMR spectrum of the compound 7.(b) 13C NMR spectrum of the compound 7. (TIF) [file pone.0156129.s007.tif]

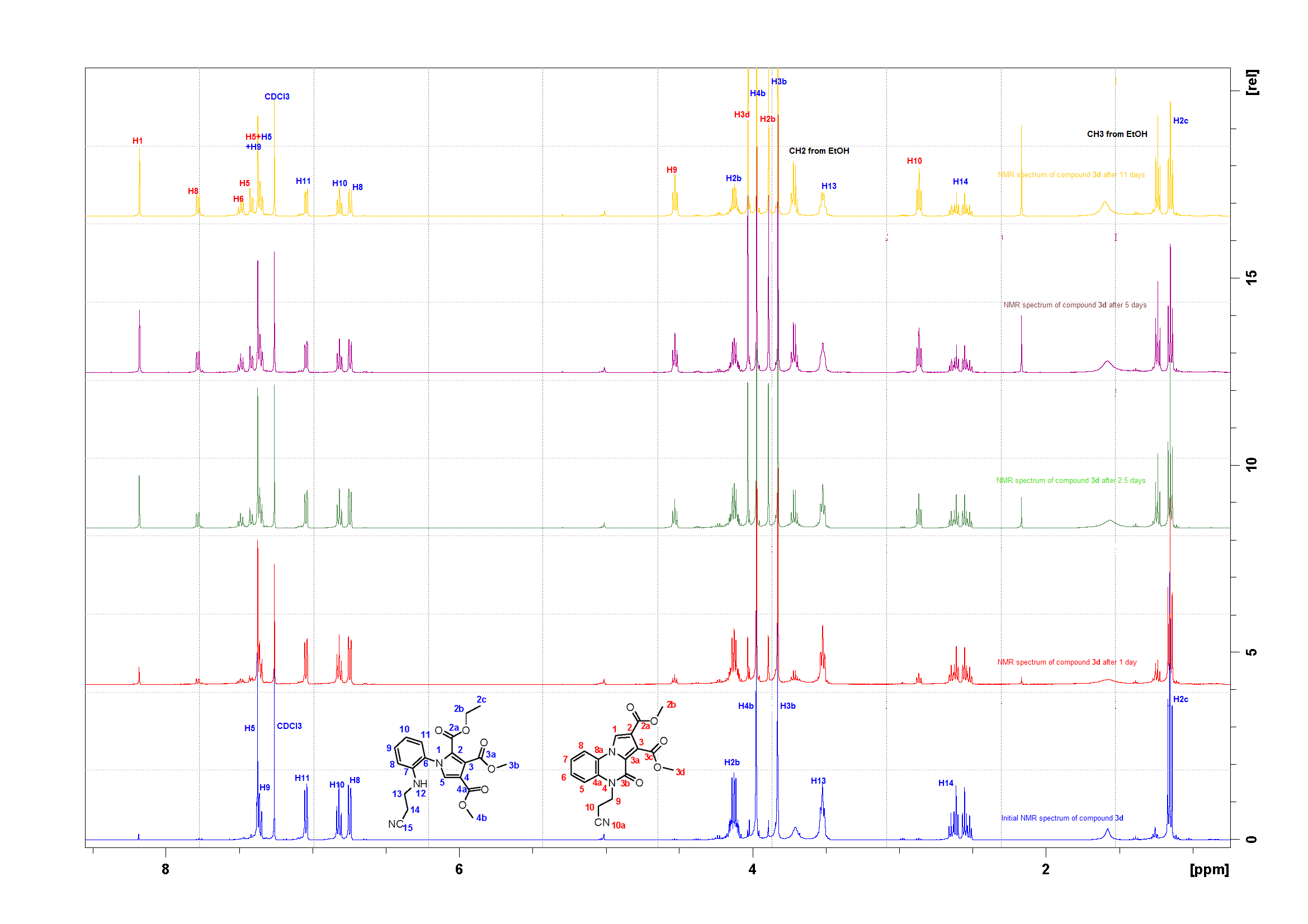

Supplement: S6 Fig — (TIFF) [file pone.0156129.s008.tiff]
