# Supplementary material for: The Cycloaddition of the Benzimidazolium Ylides with Alkynes: New Mechanistic Insights
Source: PLoS One. 2016 May 25;11(5):e0156129. doi: 10.1371/journal.pone.0156129 (PMC4880325; doi:10.1371/journal.pone.0156129)
Supplement: S1 File — Spectral characterization, NMR spectra (1H and 13C) of the obtained compounds, and 1H-NMR studies on 4b at room temperature. (DOCX) [file pone.0156129.s009.docx]

**Supporting information for:**

**The cycloaddition of the benzimidazolium ylides with alkynes: new mechanistic insights.**

**Costel Moldoveanu, Gheorghita Zbancioc, Dorina Mantu, Dan Maftei and Ionel Mangalagiu**

Departament of Chemistry, Alexandru Ioan Cuza University of Iasi, Iasi, Romania.

^*^Corresponding author

E-mail: gheorghita.zbancioc@uaic.ro (GZ)

Contents

[1. Spectral characterization of the obtained compounds. 2](#_Toc451154817)

[1.1: Trimethyl 1-(2-(2-cyanoethylamino)phenyl)-1H-pyrrole-2,3,4-tricarboxylate (**4a**). 2](#_Toc451154818)

[1.2: 2-Ethyl 3,4-dimethyl 1-(2-(2-cyanoethylamino)phenyl)-1H-pyrrole-2,3,4-tricarboxylate (**4b**). 2](#_Toc451154819)

[1.3: Dimethyl 5-(2-cyanoethyl)-4-oxo-4,5-dihydropyrrolo[1,2-a]quinoxaline-2,3-dicarboxylate (**5**). 2](#_Toc451154820)

[1.4: Dimethyl 5-(3-methoxy-3-oxopropyl)-4-oxo-4,5-dihydropyrrolo[1,2-a]quinoxaline-2,3-dicarboxylate (**6**). 2](#_Toc451154821)

[1.5: Dimethyl 5-(3-ethoxy-3-oxopropyl)-4-oxo-4,5-dihydropyrrolo[1,2-a]quinoxaline-2,3-dicarboxylate (**7**). 3](#_Toc451154822)

[2. NMR Spectra of the obtained compounds. 4](#_Toc451154823)

[3. ^1^H-NMR studies on compound 4b at room temperature. 9](#_Toc451154824)

- 1. Spectral characterization of the obtained compounds.

The following abbreviations were used to designate chemical shift multiplicities: s = singlet, d = doublet, dd = doublet of doublet, t = triplet, m = multiplet. Chemical shifts were reported in delta (δ) units, part per million (ppm) and coupling constants (*J*) in Hz.

1.1: Trimethyl 1-(2-(2-cyanoethylamino)phenyl)-1H-pyrrole-2,3,4-tricarboxylate (**4a**).

White crystals, mp 232-234°C. R_f_ (98/2 CH_2_Cl_2_/CH_3_OH) 0.38. IR (cm^-1^): 3350, 3136 (NH, amine), 3048, 3023 (C-H arom.), 2955, 2920 (C-H aliph.), 2247 (C≡N) 1736, 1705 (C=O, ester), 1609, 1547, 1523, 1458, 1437, 1396 (aromatic and heteroaromatic ring), 1294, 1274, 1251, 1231, 1165, 1140 1067 (C–O–C, ester), 748 (ortho disubstituted aromatic). ^1^H NMR (500 MHz, CDCl_3_): *δ* 7.38 (1H, s, overlapped peaks, H-5), 7.36 (1H, td, overlapped peaks, H-9), 7.04 (1H, dd, *J*= 7.5, 1.5 Hz, H-11), 6.81 (1H, td, *J*= 7.5, 1.5 Hz, H-10), 6.74 (1H, d, *J*= 8.0 Hz, H-8), 3.97 (3H, s, C-4b-H), 3.82 (3H, s, C-3b-H), 3.68 (3H, s, C-2b-H), 3.51 (2H, q, *J*= 7.0 Hz, H-13), 2.57 (2H, m, *J*= 7.0 Hz, H-14). ^13^C NMR (125 MHz, CDCl_3_): *δ* 165.89 (CO of C-4a), 162.71 (CO of C-3a), 158.89 (CO of C-2a), 142.38 (C-7), 132.29 (C-5), 130.87 (C-9), 128.27 (C-11), 125.81 (C-6), 125.74 (C-2), 122.18 (C-4), 118.10 (C-10), 117.80 (C-15), 115.49 (C-3), 111.25 (C-8), 53.06 (C-4b), 52.25 (C-2b), 51.95 (C-3b), 39.28 (C-13), 17.88 (C-14).

1.2: 2-Ethyl 3,4-dimethyl 1-(2-(2-cyanoethylamino)phenyl)-1H-pyrrole-2,3,4-tricarboxylate (**4b**).

Yellow crystals, mp 126-128°C. R_f_ (98/2 CH_2_Cl_2_/CH_3_OH) 0.36. IR (cm^-1^): 3399, 3132 (NH, amine), 3075 (C-H arom.), 2996, 2949 (C-H aliph.), 2247 (C≡N) 1734, 1712 (C=O, ester), 1610, 1587, 1527, 1462, 1438, 1410 (aromatic and heteroaromatic ring), 1298, 1249, 1222, 1134, 1116, 1070 (C–O–C, ester), 750 (ortho disubstituted aromatic). ^1^H NMR (500 MHz, CDCl_3_): *δ* 7.37 (1H, s, overlapped peaks, H-5), 7.35 (1H, t, overlapped peaks, H-9), 7.04 (1H, dd, *J*= 6.5, 1.0 Hz, H-11), 6.81 (1H, t, *J*= 6.5, 1.0 Hz, H-10), 6.74 (1H, d, *J*= 8.0 Hz, H-8), 4.12 (2H, q, *J*= 7.0 Hz, H-2b), 3.96 (3H, s, C-4b-H), 3.82 (3H, s, C-3b-H), 3.51 (2H, t, *J*= 7.0 Hz, H-13), 2.58 (2H, m, *J*= 7.0 Hz, H-14), 1.14 (3H, t, *J*= 7.0 Hz, H-2c). ^13^C NMR (125 MHz, CDCl_3_): *δ* 165.89 (CO of C-4a), 162.74 (CO of C-3a), 158.42 (CO of C-2a), 142.42 (C-7), 132.11 (C-5), 130.81 (C-9), 128.31 (C-11), 125.86 (C-6), 125.79 (C-4), 122.49 (C-3), 118.06 (C-10), 117.77 (C-15), 115.45 (C-2), 111.20 (C-8), 61.19 (C-2b), 52.94 (C-4b), 51.92 (C-3b), 39.32 (C-13), 17.90 (C-14), 13.88 (C-2c).

1.3: Dimethyl 5-(2-cyanoethyl)-4-oxo-4,5-dihydropyrrolo[1,2-a]quinoxaline-2,3-dicarboxylate (**5**).

White crystals, mp 232-233°C. R_f_ (98/2 CH_2_Cl_2_/CH_3_OH) 0.30. IR (cm^-1^): 3067, 3015 (C-H arom.), 2986, 2955 (C-H aliph.), 2253 (C≡N), 1728, 1710 (C=O, ester), 1659 (C=O, amide), 1614, 1552, 1523, 1462, 1447, 1411, 1377 (heteroaromatic ring), 1277, 1254, 1199, 1167, 1151, 1072, (C–O–C, ester). ^1^H NMR (500 MHz, CDCl_3_): *δ* 8.17 (1H, s, H-1), 7.77 (1H, dd, *J*= 7.5, 0.5 Hz, H-8), 7.48 (1H, td, *J*= 7.5, 1.0 Hz, H-6), 7.42 (1H, d, *J*= 7.5 Hz, H-5), 7.35 (1H, td, *J*= 7.5, 0.5 Hz, H-7), 4.53 (2H, q, *J*= 7.0 Hz, H-9), 4.03 (3H, s, C-3d-H), 3.89 (3H, s, C-2b-H), 2.86 (2H, t, *J*= 7.0 Hz, H-10). ^13^C NMR (125 MHz, CDCl_3_): *δ* 165.25 (CO of C-3c), 162.76 (CO of C-2a), 154.09 (CO of C-3b), 129.10 (C-4a), 128.02 (C-6), 124.44 (C-7), 122.85 (C-8a), 121.61 (C-3), 120.34 (C-3a), 119.27 (C-1), 118.21 (C-2), 117.00 (C-10a), 116.00 (C-8), 115.55 (C-5), 53.27 (C-3d), 52.30 (C-2b), 37.71 (C-9), 16.25 (C-10).

1.4: Dimethyl 5-(3-methoxy-3-oxopropyl)-4-oxo-4,5-dihydropyrrolo[1,2-a]quinoxaline-2,3-dicarboxylate (**6**).

Yellowish crystals, mp 166-167°C. R_f_ (98/2 CH_2_Cl_2_/CH_3_OH) 0.34. IR (cm^-1^): 3024, 3009 (C-H arom.), 2957 (C-H aliph.), 1736, 1711 (C=O, ester), 1661 (C=O, amide), 1613, 1564, 1521, 1448, 1431, 1416, 1377 (heteroaromatic ring), 1305, 1267, 1192, 1163, 1138, 1069, 1047 (C–O–C, ester). ^1^H NMR (500 MHz, CDCl_3_): *δ* 8.14 (1H, s, H-1), 7.74 (1H, dd, *J*= 7.0, 1.5 Hz, H-8), 7.41 (2H, m, overlapped peaks, H-5, H-6), 7.29 (1H, td, *J*= 7.0, 1.5 Hz, H-7), 4.51 (2H, t, *J*= 8.0 Hz, H-9), 4.02 (3H, s, C-3d-H), 3.87 (3H, s, C-2b-H), 3.69 (3H, s, C-10b-H), 2.76 (2H, t, *J*= 8.0 Hz, H-10). ^13^C NMR (125 MHz, CDCl_3_): *δ* 171.44 (CO of C-10a), 165.46 (CO of C-3c), 162.90 (CO of C-2a), 154.05 (CO of C-3b), 129.43 (C-4a), 127.83 (C-6), 123.83 (C-7), 122.80 (C-8a), 121.01 (C-3), 120.79 (C-3a), 118.86 (C-1), 117.89 (C-2), 115.82 (C-5), 115.70 (C-8), 53.19 (C-3d), 52.20 (C-2b), 52.14 (C-10b), 37.56 (C-9), 31.94 (C-10).

1.5: Dimethyl 5-(3-ethoxy-3-oxopropyl)-4-oxo-4,5-dihydropyrrolo[1,2-a]quinoxaline-2,3-dicarboxylate (**7**).

White crystals, mp 164-165°C. R_f_ (98/2 CH_2_Cl_2_/CH_3_OH) 0.34. IR (cm^-1^): 3065, 3016 (C-H arom.), 2986, 2947 (C-H aliph.), 1730, 1711 (C=O, ester), 1667 (C=O, amide), 1616, 1564, 1524, 1437, 1416, 1370 (heteroaromatic ring), 1308, 1271, 1256, 1240, 1198, 1161, 1142, 1078, (C–O–C, ester). ^1^H NMR (500 MHz, CDCl_3_): *δ* 8.16 (1H, s, H-1), 7.75 (1H, d, *J*= 8.0 Hz, H-8), 7.44 (2H, m, overlapped peaks, H-5, H-6), 7.31 (1H, td, *J*= 8.0, 1.5 Hz, H-7), 4.53 (2H, t, *J*= 8.0 Hz, H-9), 4.16 (2H, q, *J*= 7.5 Hz, H-10b), 4.04 (3H, s, C-3d-H), 3.89 (3H, s, C-2b-H), 2.77 (2H, t, *J*= 8.0 Hz, H-10), 1.24 (3H, t, *J*= 7.5 Hz, H-10c). ^13^C NMR (125 MHz, CDCl_3_): *δ* 171.06 (CO of C-10a), 165.50 (CO of C-3c), 162.95 (CO of C-2a), 154.11 (CO of C-3b), 129.55 (C-4a), 127.86 (C-6), 123.84 (C-7), 122.87 (C-8a), 121.06 (C-3), 120.89 (C-3a), 118.85 (C-1), 117.87 (C-2), 115.95 (C-5), 115.70 (C-8), 61.17 (C-10b), 53.23 (C-3d), 52.24 (C-2b), 37.64 (C-9), 32.23 (C-10), 14.27 (C-10c).

- 1. NMR Spectra of the obtained compounds.


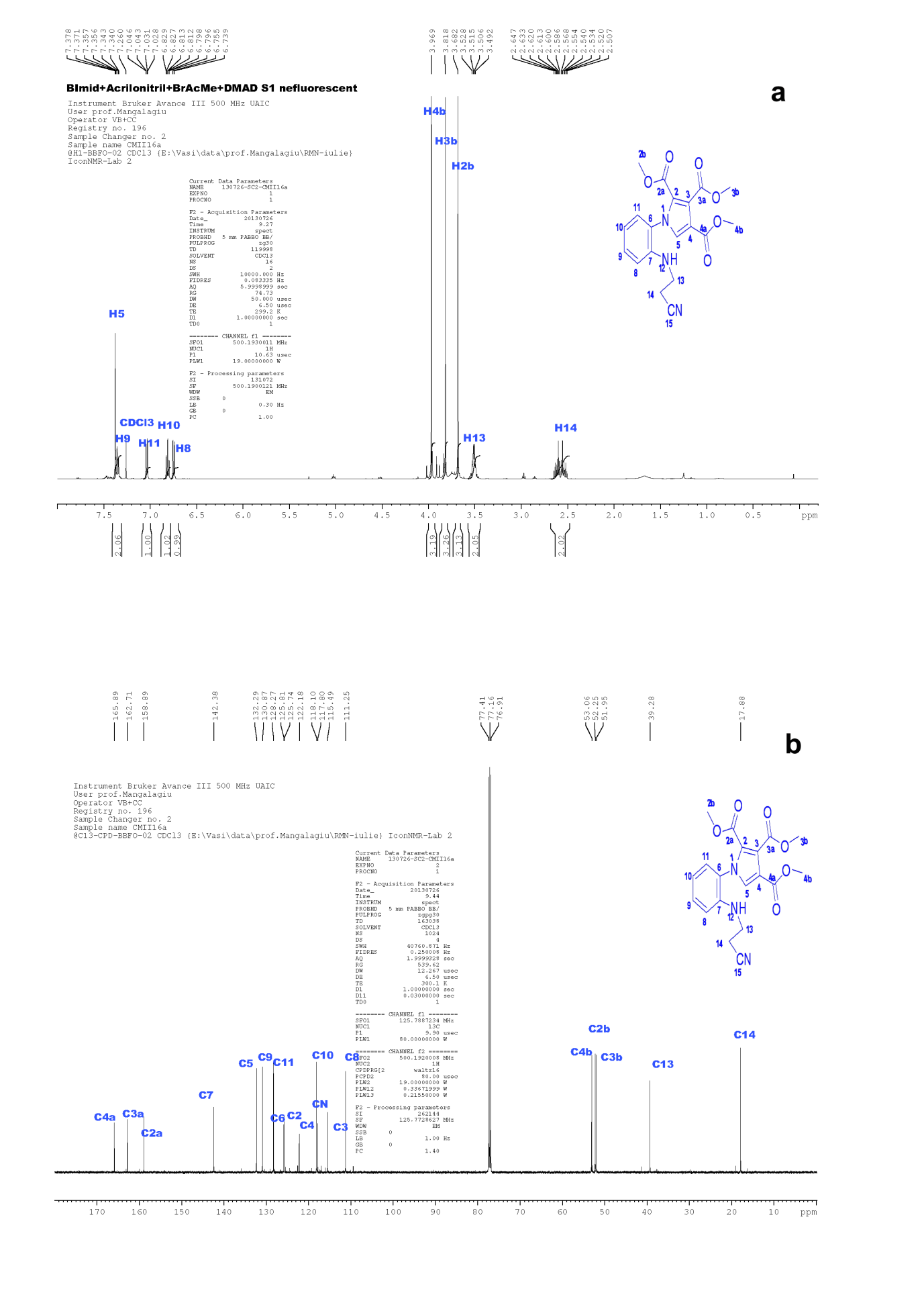


**S1 Fig - (a)** ^1^H NMR spectrum of the compound **4a. (b)** ^13^C NMR spectrum of the compound **4a.**

*
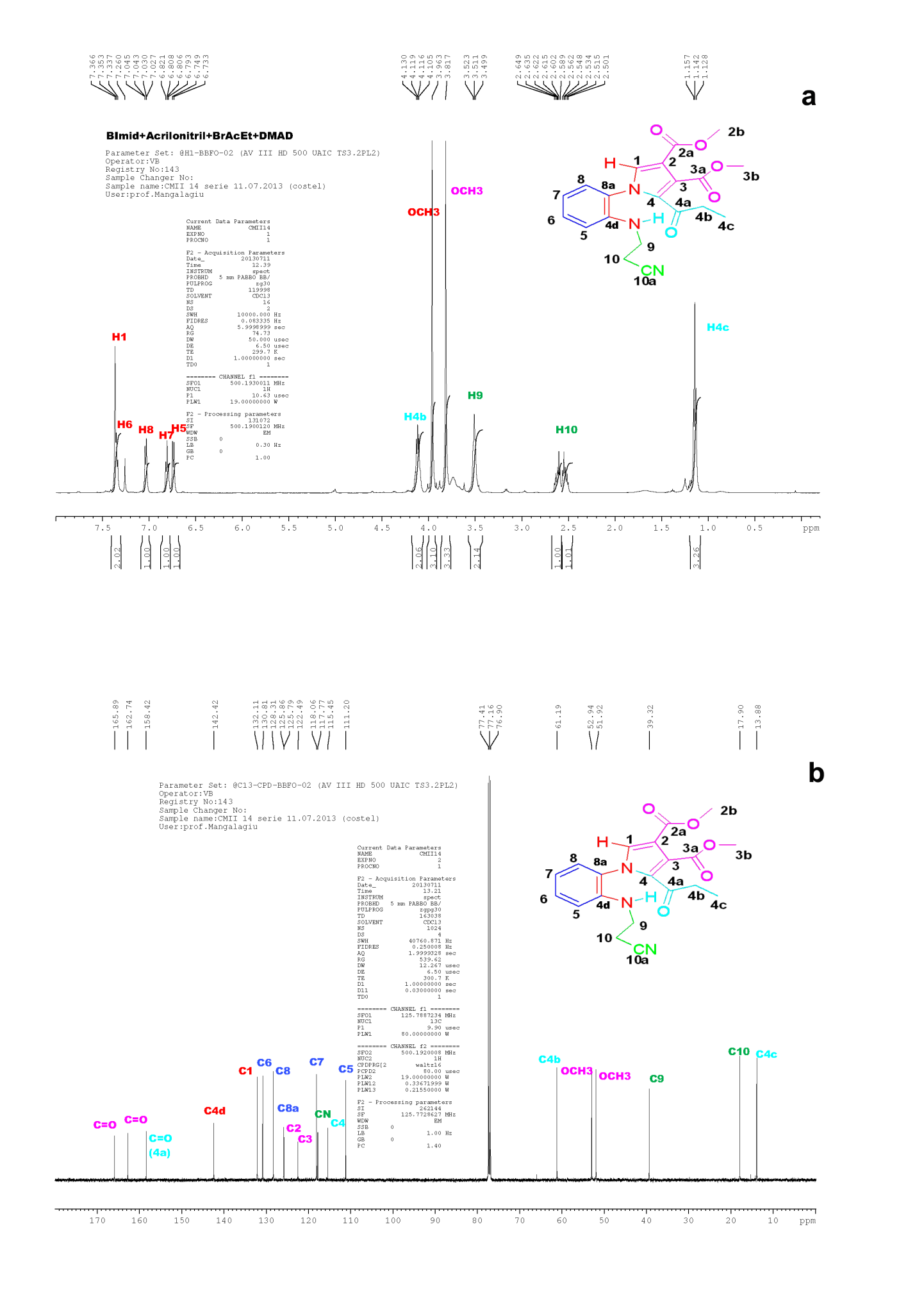
*

**Fig S2 - (a)** ^1^H NMR spectrum of the compound **4b. (b)** ^13^C NMR spectrum of the compound **4b.**

*
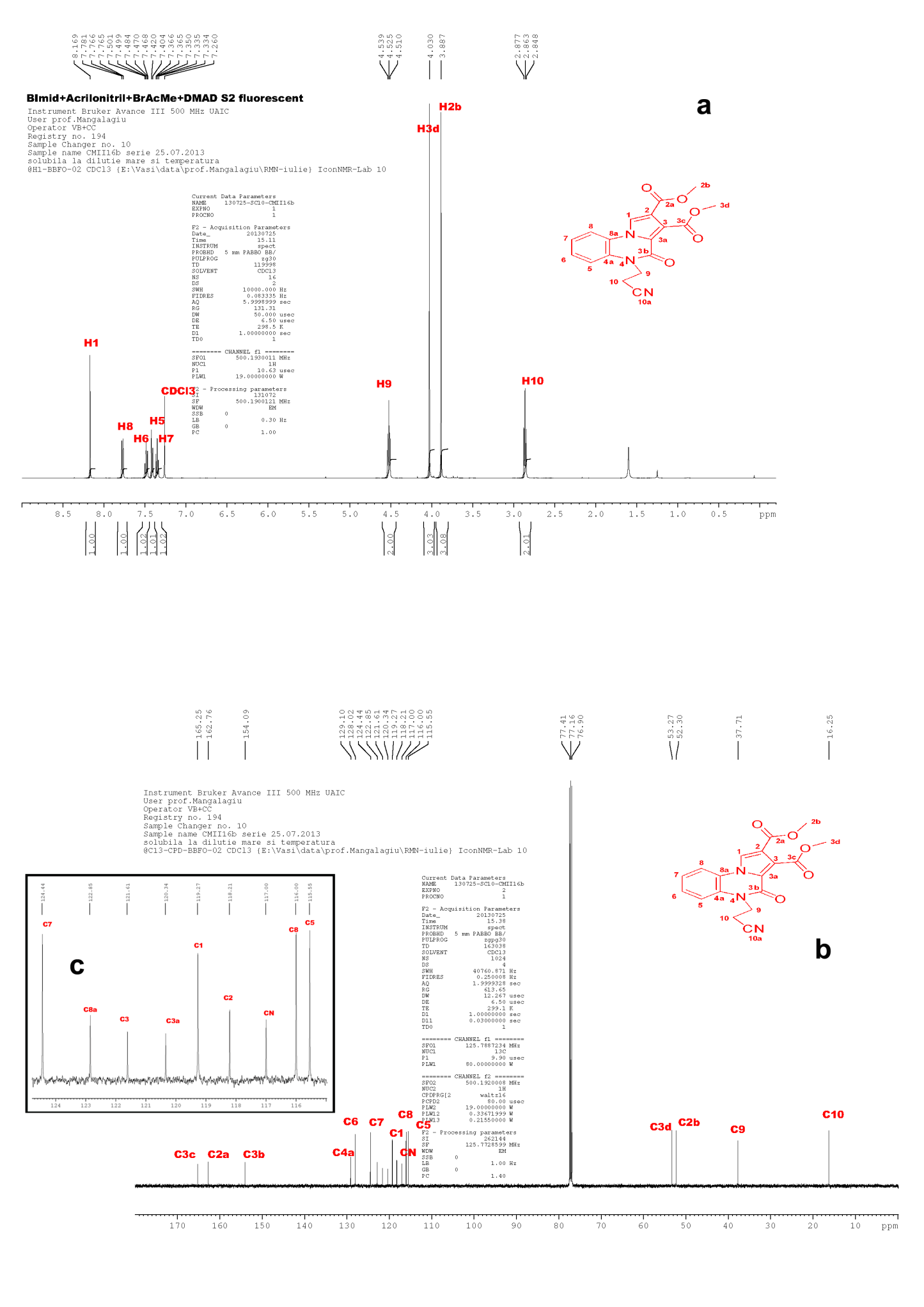
*

**S3 Fig - (a)** ^1^H NMR spectrum of the compound **5. (b)** ^13^C NMR spectrum of the compound **5. (c).** Detail on ^13^C NMR spectrum of the compound **5.**

**c**

***
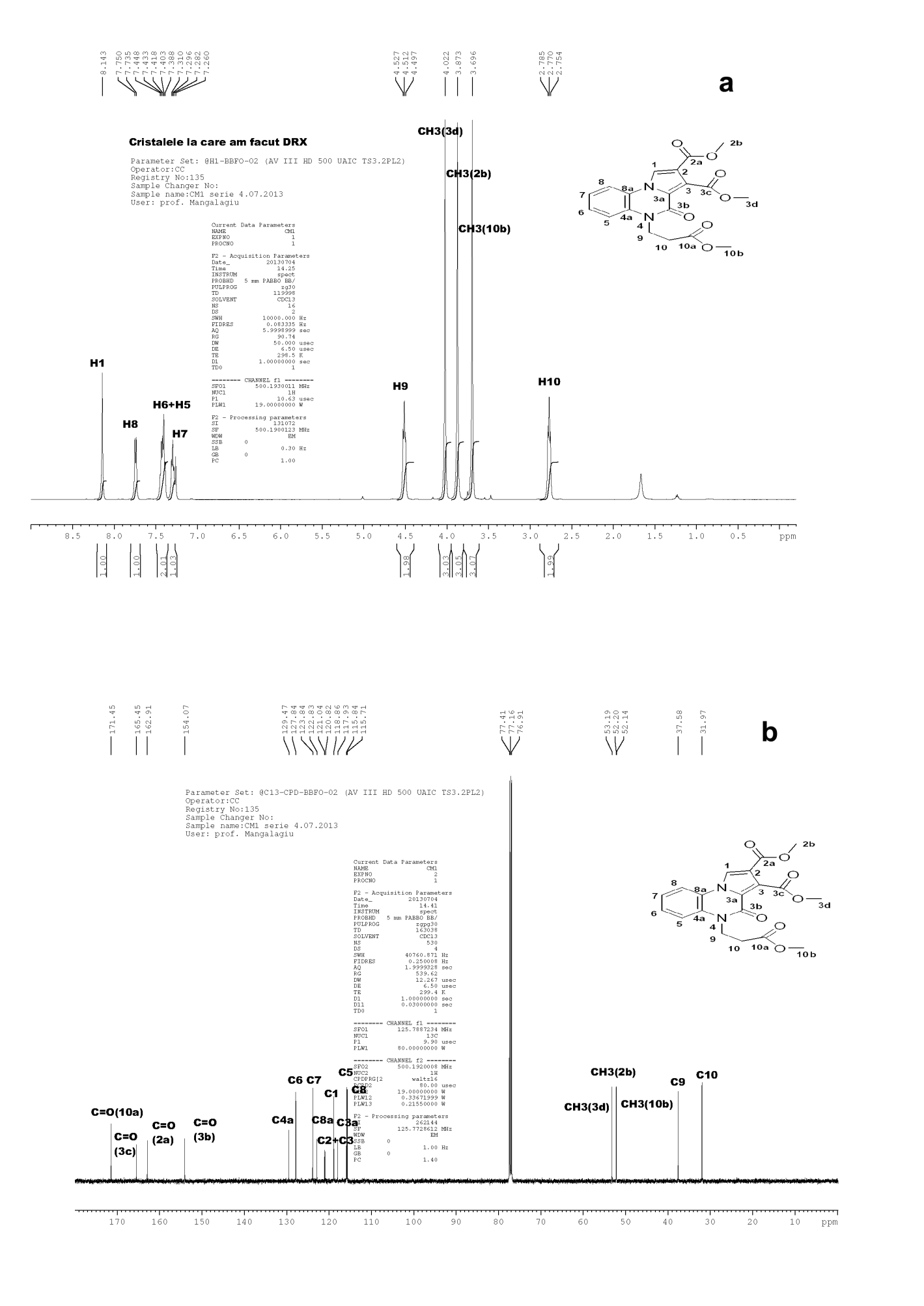
***

**S4 Fig - (a)** ^1^H NMR spectrum of the compound **6. (b)** ^13^C NMR spectrum of the compound **6.**

***

***

**S5 Fig - (a)** ^1^H NMR spectrum of the compound **7. (b)** ^13^C NMR spectrum of the compound **7.**

- 1. ^1^H-NMR studies on compound 4b at room temperature.

*NMR sample preparation:*

10 mg of 2-ethyl 3,4-dimethyl 1-(2-(2-cyanoethylamino)phenyl)-1H-pyrrole-2,3,4-tricarboxylate (**4b**) were dissolved in 1 mL CDCl_3_ and inserted in a 5 mm diameter NMR tube and sealed with a polypropylene cap. A ^1^H-NMR spectrum was quickly recorded than the sample was stored at room temperature. After 1 day, 2.5 days, 5 days and 11 days respectively a new ^1^H-NMR spectrum was recorded for the sample.

The ^1^H-NMR spectra are presented in the S6 Fig


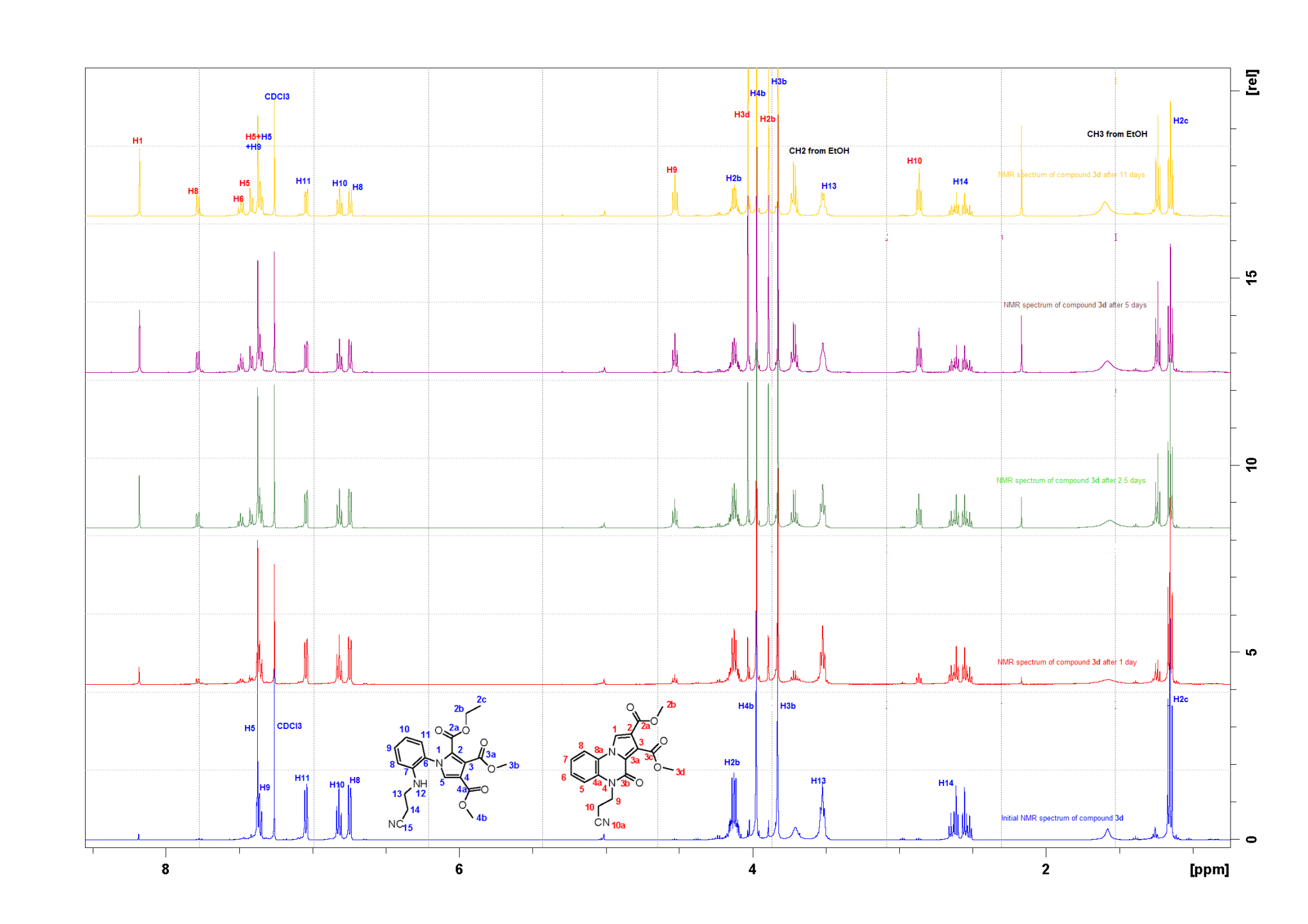


**S6 Fig.** Time evolution of the ^1^H-NMR spectrum of 2-ethyl 3,4-dimethyl 1-(2-(2-cyanoethylamino)phenyl)-1H-pyrrole-2,3,4-tricarboxylate (**4b**).
